# P22-Based Nanovaccines against Enterohemorrhagic Escherichia coli

**DOI:** 10.1128/spectrum.04734-22

**Published:** 2023-03-21

**Authors:** Alejandro Huerta-Saquero, Itziar Chapartegui-González, Sarah Bowser, Nittaya Khakhum, Jacob L. Stockton, Alfredo G. Torres

**Affiliations:** a Centro de Nanociencias y Nanotecnología, Universidad Nacional Autónoma de México, Ensenada, Baja California, México; b Department of Microbiology and Immunology, University of Texas Medical Branch, Galveston, Texas, USA; Cinvestav-IPN

**Keywords:** pathogenic *Escherichia coli*, EHEC, P22-based nanovaccines, nanocontainer

## Abstract

Enterohemorrhagic Escherichia coli (EHEC) is an important causative agent of diarrhea in humans that causes outbreaks worldwide. Efforts have been made to mitigate the morbidity and mortality caused by these microorganisms; however, the global incidence is still high, causing hundreds of deaths per year. Several vaccine candidates have been evaluated that demonstrate some stability and therapeutic potential but have limited overarching effect. Virus-like particles have been used successfully as nanocontainers for the targeted delivery of drugs, proteins, or nucleic acids. In this study, phage P22 nanocontainers were used as a carrier for the highly antigenic T3SS structural protein EscC that is conserved between EHEC and other enteropathogenic bacteria. We were able to stably incorporate the EscC protein into P22 nanocontainers. The EscC-P22 particles were used to intranasally inoculate mice, which generated specific antibodies against EscC. These antibodies increased the phagocytic activity of murine macrophages infected with EHEC *in vitro* and reduced bacterial adherence to Caco-2 epithelial cells *in vitro*, illustrating their functionality. The EscC-P22-based particles are a potential nanovaccine candidate for immunization against EHEC O157:H7 infections.

**IMPORTANCE** This study describes the initial attempt to use P22 viral-like particles as nanocontainers expressing enterohemorrhagic Escherichia coli (EHEC) proteins that are immunogenic and could be used as effective vaccines against EHEC infections.

## INTRODUCTION

Escherichia coli is a large and diverse group of Gram-negative bacteria. While there are environmental and commensal strains, others are pathogenic, like those belonging to the enterohemorrhagic E. coli (EHEC) pathovar, which represents an important human pathogen that colonizes the gut mucosa and produces attaching and effacing (A/E) lesions (1). A/E lesions are characterized by intimate bacterial attachment to the apical side of the intestinal membrane, with the subsequent localized accumulation of F-actin and effacement of the brush border microvilli (2). Ruminants are a reservoir for EHEC, and the ingestion of contaminated beef or green products can result in outbreaks, which can pose a risk to human health. The Centers for Disease Control and Prevention has developed a surveillance program (https://www.cdc.gov/ecoli/outbreaks.html) to monitor these outbreaks, with EHEC serotype O157:H7 being the most common cause.

Infections with the emerging zoonotic pathogen EHEC can range from acute gastroenteritis and self-resolving diarrheal episodes to hemorrhagic colitis, mainly in children younger than 5 years old and the elderly (3), which can progress to hemolytic uremic syndrome (HUS) (4, 5). HUS development is the result of the translocation of Shiga toxins (Stx1 and Stx2) across the gut, leading to kidney failure and chronic postinfection sequelae or death ([Bibr B6][Bibr B7] 8). In addition to the intoxication, EHEC adheres to the intestinal epithelia using fimbriae and/or pili, colonizing the intestinal mucosa (4, 5, 9 to 11). After the initial contact, EHEC establishes intimate attachment to the surface of the intestinal epithelial cells (IECs) by the translocation of virulence factors through the type 3 secretion system (T3SS) apparatus. The injected virulence factors are responsible for the formation of A/E lesions on IECs, forming a “cup-like structure” (4). The genes encoding the T3SS proteins and several virulence factors are localized in the locus of enterocyte effacement (LEE) pathogenicity island, which is absent in nonpathogenic E. coli strains (12).

There are no specific treatments to combat pathogenic E. coli infections, and in the case of EHEC strains, the use of antibiotics promotes the production of toxins (Stx) (13, 14). This process induces bacterial lysis, allowing the release and dissemination of Stx into the gastrointestinal tract and other distal organs (15). Although antibiotic treatment is usually effective against other intestinal pathotypes of E. coli, overuse of them has created multidrug-resistant strains, reflecting an important public health concern (16, 17). This highlights the need for new strategies to control infections caused by these pathogens, and the development of prophylactic vaccines is not only necessary but a viable alternative to prevent infections ([Bibr B18][Bibr B19] 20).

Previous attempts at vaccine development have been mainly based on the use of proteins encoded in the LEE pathogenicity island associated with adhesion or translocation, such as the intimin adhesin and the T3SS component EspA (21), as well as the Shiga toxin subunits (22), and bacterial ghost cells derived from EHEC O157:H7 (23), among others. These vaccine formulations have demonstrated variable success in murine models of infection (reviewed in references [Bibr B8][Bibr B9][Bibr B10][Bibr B11][Bibr B12][Bibr B13][Bibr B14][Bibr B15][Bibr B16][Bibr B17][Bibr B18][Bibr B19] 20). In searching for novel vaccine production platforms, nanovaccines based on gold nanoparticles (AuNPs) have also been recently developed in which AuNPs were functionalized with bacterial antigens to elicit immune responses (24, 25). Sánchez-Villamil et al. reported the construction of a successful AuNP vaccine able to induce protective immune responses against EHEC (24, 25). The use of nanoparticles and other nanomaterials represents many advantages, such as the stabilization of proteins or other molecules that adhere to their surface (26, 27), the catalysis of physicochemical events such as oxidation or the reactive oxygen species formation, and most importantly, their safety (28, 29).

In this work, we explore the use of virus-like particles (VLPs) as potential vaccine platforms. VLPs are protein-based, self-assembling, cage architectures considered a successful model for nanoreactors and drug delivery systems (30). VLPs consist of multiple copies of self-assembling proteins, which form symmetric, stable, polyvalent, and monodisperse nanocontainers that, due to their lack of genetic material, cannot cause infections. Their self-assembly properties allow for the formation of a nanoparticle identical to the original virus capsid, but instead of genetic material, contains a molecule of interest, such as biomolecules or drugs. The application of these systems has been widely studied and represents an innovative approach in human medicine ([Bibr B30][Bibr B31][Bibr B32] 33). VLPs can be used for the development of vaccines, cell imaging, drug delivery, and gene therapy, as well as for *in vitro* diagnostic methods (34). Other advantages of VLPs are their long half-life and biocompatibility, and that they can be chemically functionalized and produced in large quantities in a short time (35, 36).

VLPs of the phage P22 are one of the most studied and applied to systems for enzymatic nanoreactors because of their large payload volume and mechanically robust capsid. P22 VLPs are composed of 420 coat proteins (CPs) that assemble with the aid of scaffold proteins (SPs) to form icosahedral capsids with a diameter of 60 nm (37). The *in vivo* encapsulation of a cargo protein can be engineered by fusing the gene coding for SP with the gene coding for the protein of interest, like that of an immunogenic antigen ([Bibr B38][Bibr B39] 40). For example, the P22 system has been successfully used to incorporate protective antigens against human pathogens such as influenza virus and respiratory syncytial virus (RSV) (41, 42).

In this study, P22 VLPs were used as a carrier for highly antigenic EHEC proteins that are conserved between EHEC and other enteropathogenic bacteria, like the T3SS structural protein EscC, the putative outer membrane protein LomW, and the outer membrane protein Intimin (24). Among them, we selected the EscC-P22 nanovaccine (Nano-EscC) to intranasally inoculate mice, which generated specific antibodies against EscC, suggesting that the protein was released from the VLPs once inside the animal after vaccination. The presence of these antibodies also increased the phagocytic activity of murine macrophages and reduced bacterial adherence to epithelial cells *in vitro*, illustrating their functionality. Although the EscC-P22-based nanovaccine is a potential candidate for immunization against EHEC O157:H7 infections, further studies and better characterization will be needed before it can be evaluated as a protective antigen during *in vivo* experiments in suitable animal models, as well as to investigate cross-protection elicited by this vaccine against EHEC and other E. coli pathotypes. The main goal of this work was to construct stable P22 VLPs and evaluate the functionality and immunogenic characteristics of P22-based nanocontainers, which can be used as nanovaccines specifically designed for different relevant bacterial pathogens.

## RESULTS AND DISCUSSION

### *In vivo* assembly of nanovaccines.

Based on previous results that showed gold nanoparticles carrying the predicted highly antigenic EHEC EscC and LomW proteins exhibited protection in a murine model of EHEC colonization (24, 25, 43), and that intimin is a key adhesin of EHEC (9), we functionalized those antigens within P22 phage capsids to test them as potential nanovaccines.

**FIG 1:**
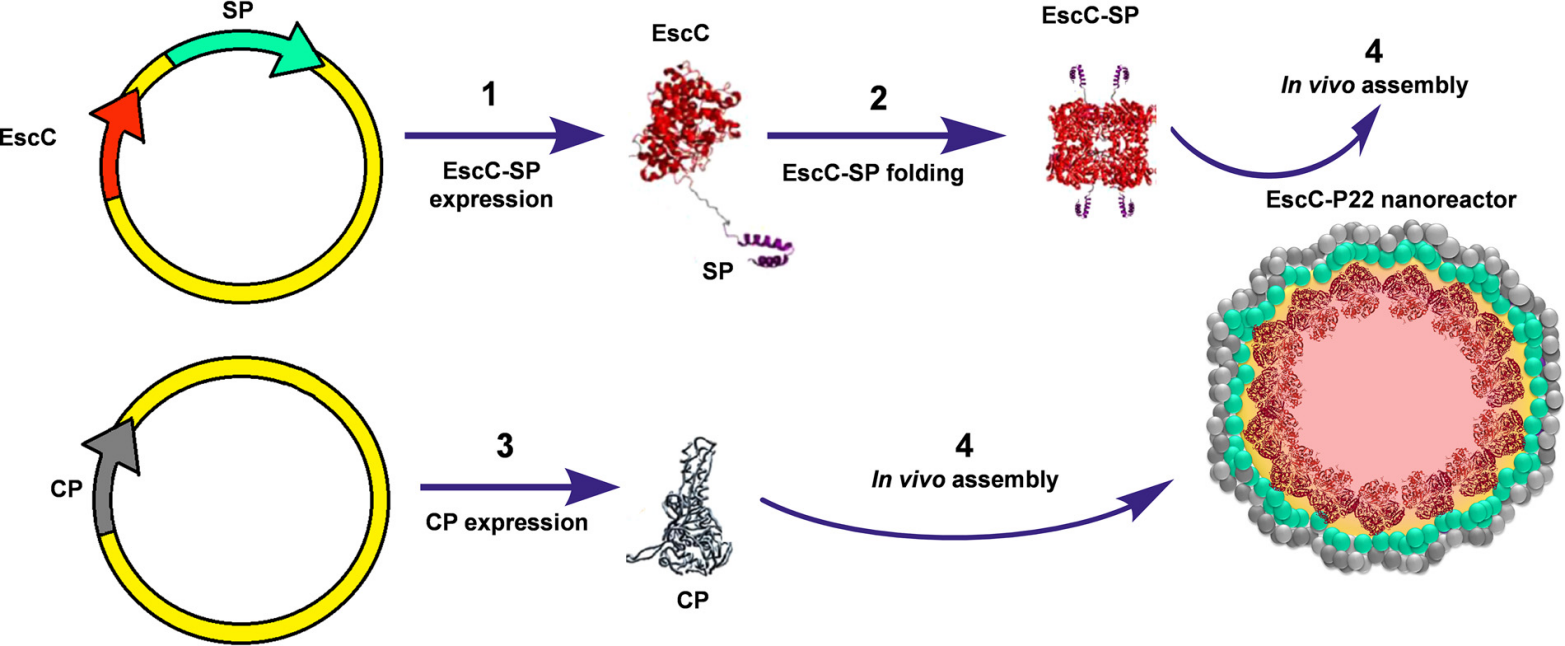
Schematic representation of P22-based nanovaccine construction (modified from reference 39).

The genes encoding the EscC, LomW, and Intimin proteins were amplified by PCR and cloned in-frame to the gene encoding the phage P22 scaffold protein (*sp* gene) into the pBAD-SP vector, to obtain the pBAD *escC-sp*, *lomW-sp*, and *eae-sp* plasmids. After obtaining cotransformant BL21 strains with pBAD *escC-sp*, pBAD *lomW-sp*, or pBAD *escC-sp*, and pRSF*-cp* plasmids, respectively, protein expression was successfully coinduced (Fig. S1 in the supplemental material). Unfortunately, the ratio of fusion proteins and capsid protein was different for each construct, with exceptionally low production of LomW-SP and Intimin-SP in soluble form, despite significant efforts to improve their yield (Fig. S2). Despite our efforts to optimize expression conditions and encapsidation of antigenic proteins (different temperatures, induction times, and inducer concentrations), the best encapsidation was achieved with the EscC-SP, with a 1:10 ratio with respect to CP, unlike LomW-SP and Intimin-SP, whose encapsidation was very reduced, obtaining a ratio less than 1:25 with respect to CP. The low proportion of these proteins in the nanocontainers led us to set them aside for further characterization so that we could focus our efforts on the production and optimization of the EscC-P22 nanovaccine. P22 capsid assembly containing the fused EscC-SP protein was optimized by first inducing EscC-SP expression for 16 h at 30°C and 180 rpm, followed by induction of CP expression for 4 h with the same culture conditions (Fig. 1).

**FIG 2:**
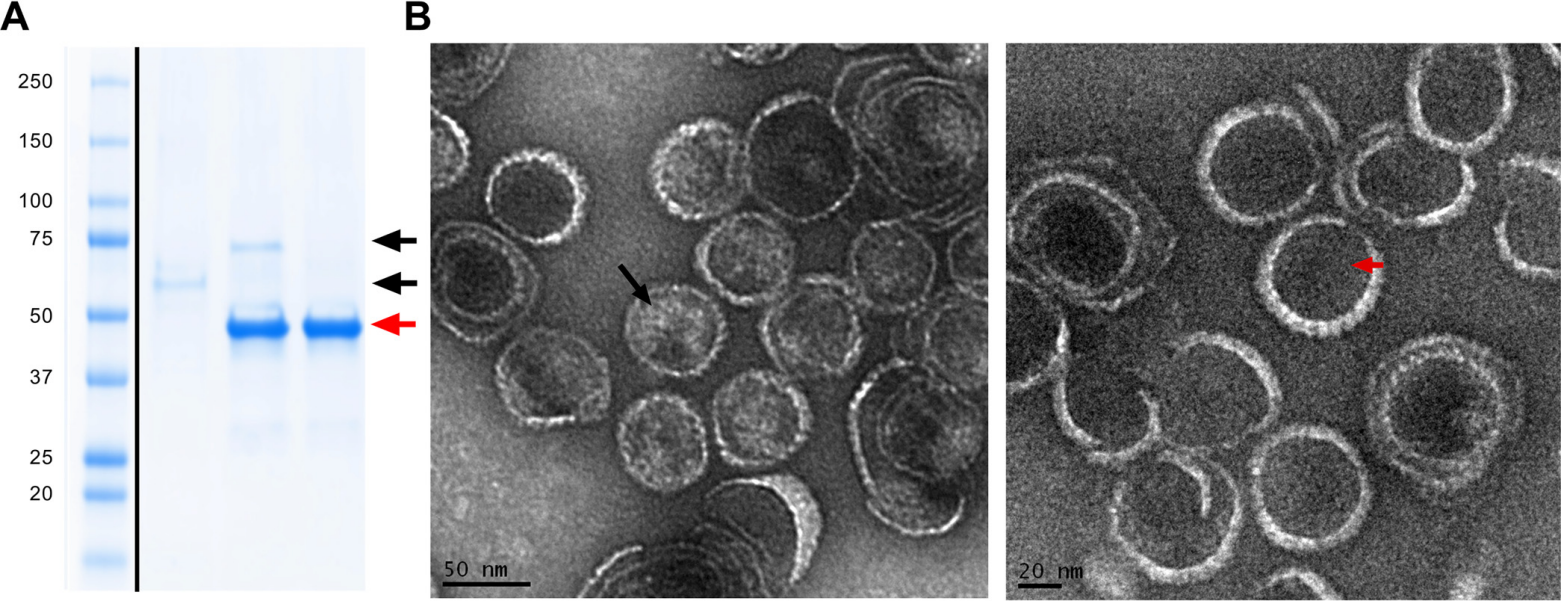
(A) SDS-PAGE showing the recombinant EscC protein (black arrows), EscC-P22 nanocontainers, and CP purified proteins (red arrow). (B) TEM micrographs of EscC-P22 nanocontainers (left) and P22 empty capsids (right). The high density inside EscC-P22 capsids (black arrow) indicates the presence of encapsulated protein, in comparison to low-density-empty capsids (red arrow).

The P22 nanocontainers harboring the EHEC-EscC protein were purified from bacterial extracts by a two-step protocol, which includes ultracentrifugation in 35% sucrose cushion followed by gel filtration chromatography. Recombinant EscC protein was purified by affinity chromatography using cobalt resin. SDS-PAGE (Fig. 2A) step-by-step verified protein expression and purification processes, and protein sequence confirmation, were obtained by mass spectrometry (Fig. S3). Purified EscC-P22 nanocontainers were characterized by SDS-PAGE followed by densitometric analysis of the protein bands, and transmission electron microscopy (TEM). EscC-P22 nanocontainer samples showed particle sizes of 62.8 ± 5.8 nm, whereas P22 empty capsids showed particle sizes of 54.3 ± 2.2 nm. We hypothesized that the VLP size variation might be the result of different amounts of encapsidated proteins (Fig. 2B).

### Mice immunization did not cause any noticeable side effects.

**FIG 3:**
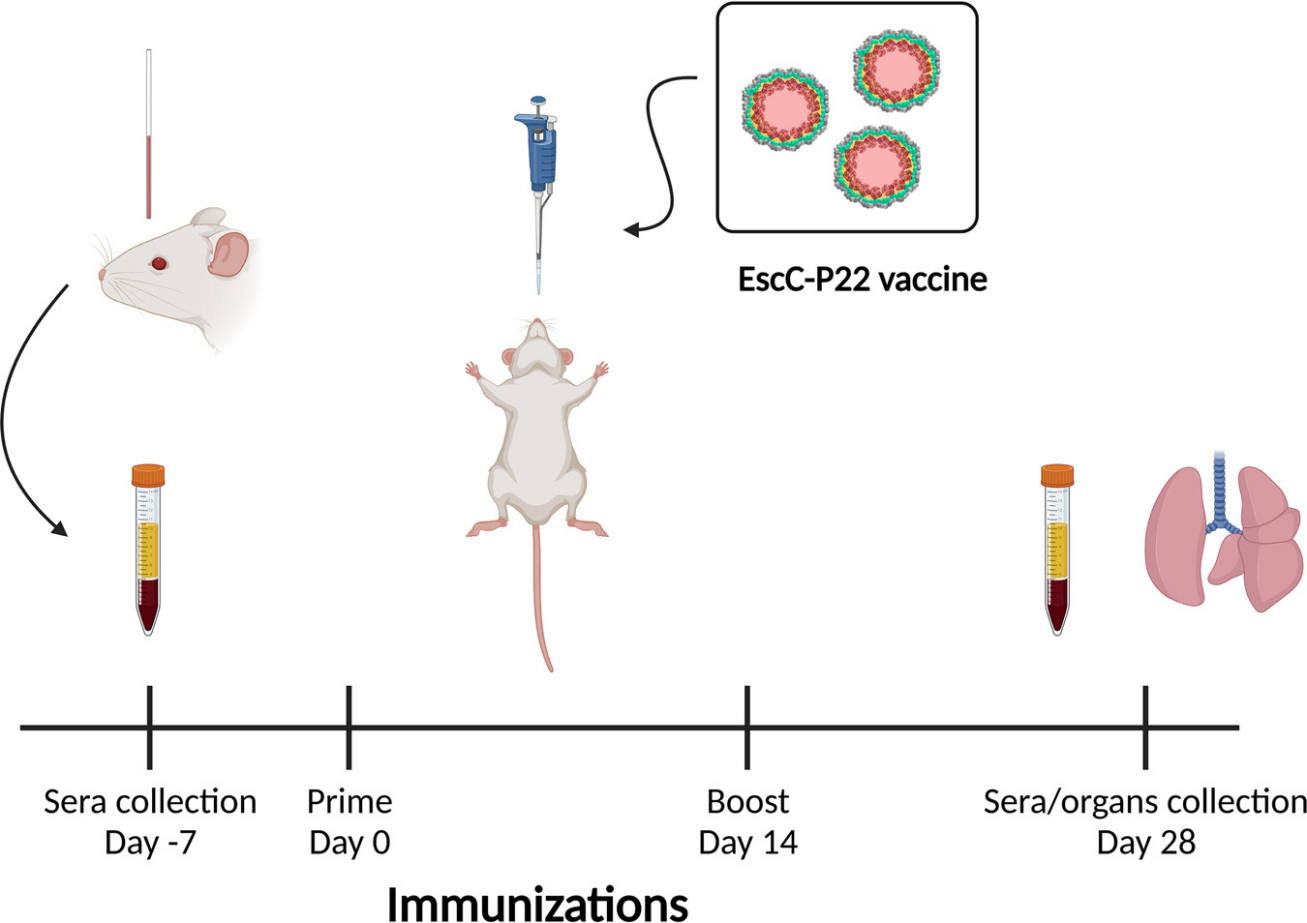
Timeline of immunization protocol. After prime and boost immunizations, sera were collected to quantify antibody production, and lungs were removed and stored for further investigation (figure created in BioRender [BioRender.com]).

To evaluate the immune response generated by the nanocontainers harboring EscC proteins (EscC-P22), female BALB/c mice were intranasally inoculated twice 2 weeks apart with EscC-P22 with and without adjuvant, or CP protein (empty capsid) as well as EscC protein with adjuvant as control groups (Fig. 3).

The physical activity and appearance of the mice were monitored after immunizations to determine any possible negative effects because of the different formulations. Both the appearance and physical activity of the mice in all groups remained unchanged, indicating that the nanovaccine did not cause any negative effects. Since the immunizations of mice were carried out intranasally, we wanted to evaluate whether the immunization could generate some damage on the lung tissue (inflammation, necrosis). For this evaluation, the lungs were obtained, and histological sections were prepared for evaluation. Initial analysis of the lung histological sections indicated that the nanovaccine produces some foci of inflammation (Fig. S4); however, further analysis is in progress to determine whether such inflammatory response is transient, because the group immunized with protein alone (EscC plus adjuvant [EscC+adj]) also showed foci of inflammation.

### Intranasal mice immunization generated immune response against EscC protein.

**FIG 4:**
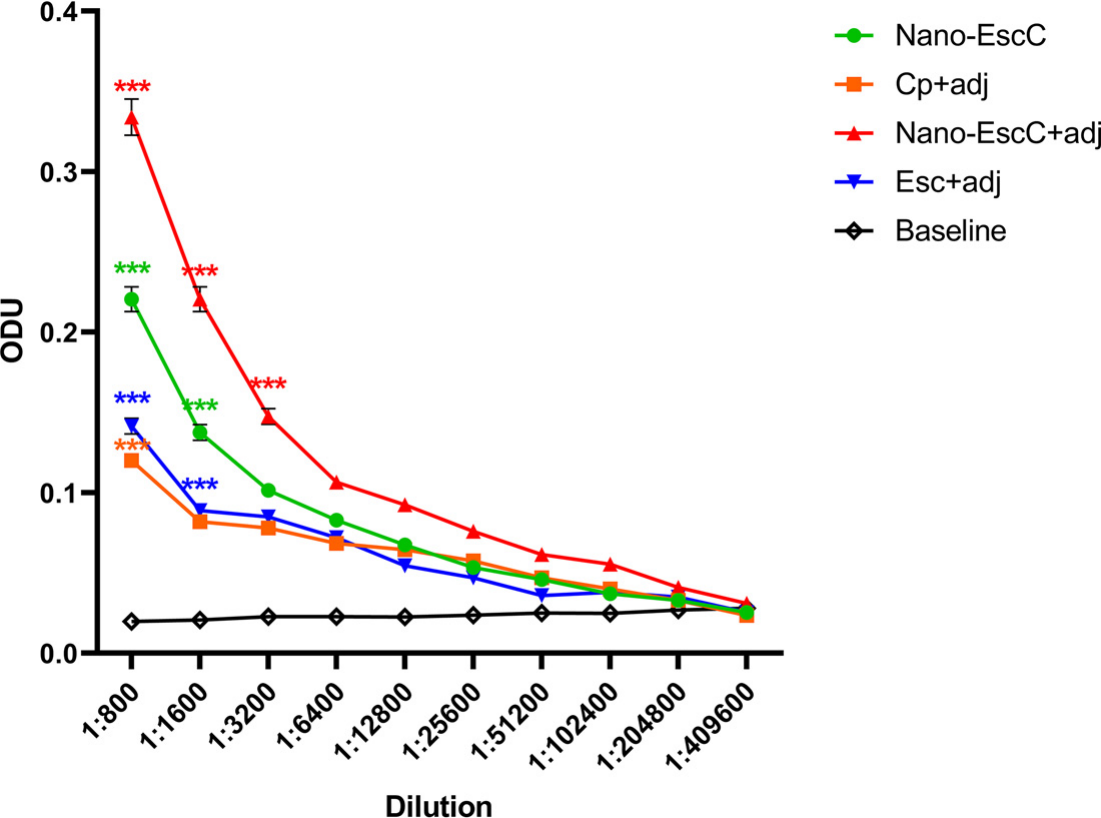
Antibody production after immunizations. ELISA was performed to measure total serum IgG titers using EscC protein as a target. Nano-EscC+adjuvant vaccine was successful in producing higher IgG titers (red line) than any other formulation, compared with preimmune sera (baseline). Significance was obtained by multiple unpaired *t* tests and shown in graph *P* values of < 0.001 (***). Statistical significance from all the dilutions can be found in Table S1.

Total IgG production was evaluated in sera of mice immunized with the different formulations. Preimmune serum samples and sera from mice inoculated with EscC-P22 nanovaccine (with and without adjuvant), as well as EscC and CP proteins, obtained after the boost immunization, were used to perform ELISAs and evaluate total IgG titers. The total IgG titers against EscC from EscC-P22 with and without adjuvant immunized mice were higher than the preimmunized mice group (baseline) (Fig. 4 and Table S1), while endpoint titers showed no significant differences (Fig. S5).

It is worth mentioning that vaccination with EscC plus adjuvant elicited a discrete immune response similar to CP plus adjuvant, but lower than that generated with the nanovaccines (Fig. 4; Nano-EscC plus or minus adjuvant [adj]). An unexpected result was the production of some IgG titers when immunization was performed with CP plus adjuvant (Fig. 4; Cp+ adj, orange line). This immune response may be due to the recognition of proteins from the E. coli BL21(DE3) used to produce the nanocontainers that might be nonspecifically interacting with the P22 capsids used for immunization, other contaminating proteins that are associated with the P22 capsids, or the capsids themselves.

### Antibodies produced by the Nano-EscC were evaluated for macrophage opsonophagocytic activity, blockage of EHEC adherence to intestinal epithelial cells, and complement-mediated bactericidal activity.

**FIG 5:**
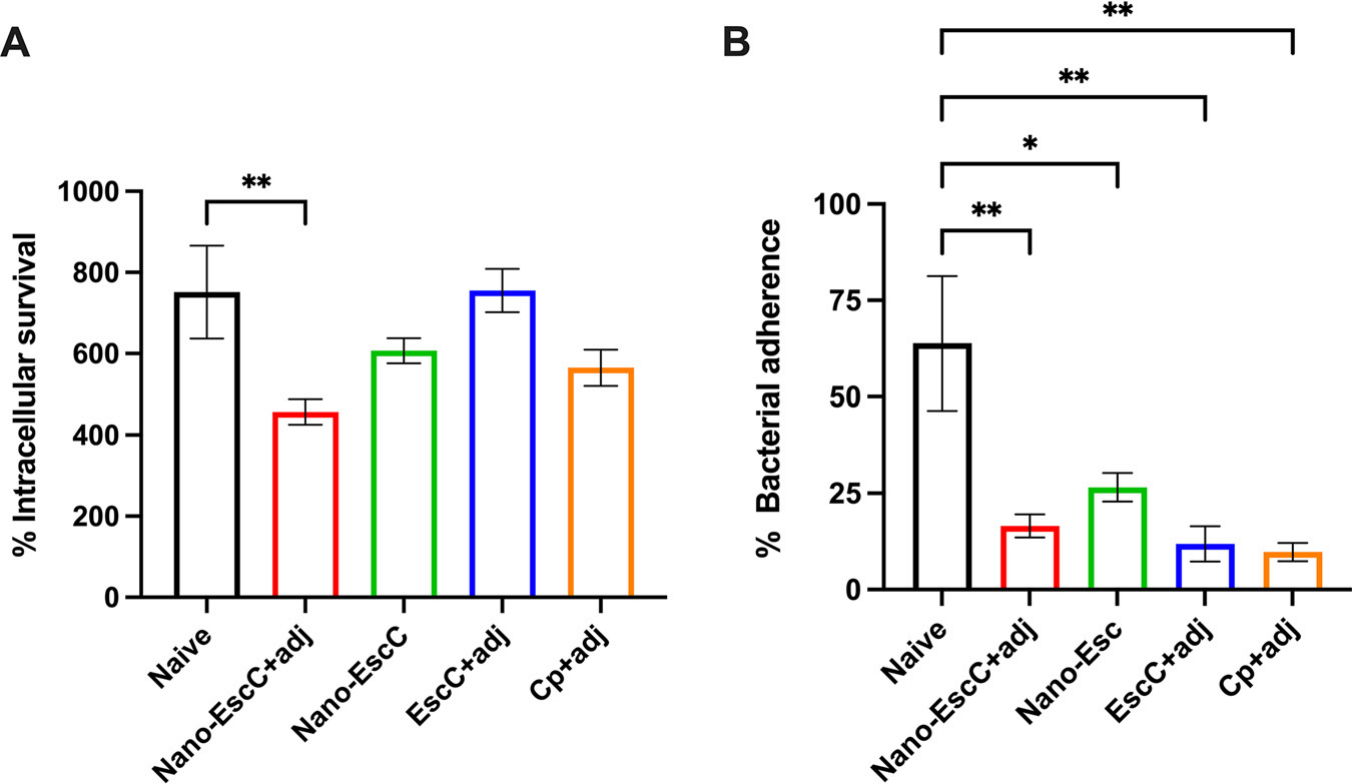
Opsonophagocytic assay (A) and bacterial adherence inhibition assay (B). (A) RAW macrophage cells were infected with EHEC O157:H7 86-24 strain previously incubated with sera obtained from vaccinated or nonvaccinated (naive) mice. Sera from nano-EscC+adj immunized mice significantly increase bacterial killing, thus reducing the recovery of internalized bacteria. The intracellular survival was established as the recovered bacteria compared with the input (×100). (B) Caco-2 epithelial cells were infected with EHEC O157:H7 86-24 strain previously incubated with sera from mice immunized with nanovaccine formulations or nonimmunized mice (naive). The antibodies reduced dramatically the bacterial adherence to epithelial cells in all the tested formulations. The percentage of adhered bacteria was established as the output bacteria compared with the inoculum (×100). (A and B) Bars represent the mean of two independent experiments performed in triplicate ± SEM (standard error). Only statistically significant data relationships are shown, from a one-way ANOVA analysis. *, *P < *0.05; **, *P < *0.01.

The functionality of the anti-EscC antibodies was analyzed in different cell lines and parameters. Opsonophagocytosis is based on the increased phagocytic capacity of macrophages by the presence of antibodies that specifically recognize the infectious agent, promoting its uptake and killing. Therefore, the generation of antibodies against EscC of EHEC could be useful for the prevention of gastrointestinal colonization by bacteria in vaccinated organisms. Incubation of bacteria with serum from Nano-EscC+adj-immunized mice promoted a significantly higher phagocytic activity by RAW 264.7 murine macrophages, which indicates a positive recognition of EHEC by the produced antibodies (Fig. 5A).

The neutralizing capacity of the antibodies produced was also verified by the inhibition of bacterial adherence to intestinal epithelial cell monolayers. The recognition of EHEC by sera antibodies was further demonstrated by the decrease in adherence of bacteria to Caco-2 epithelial cells following incubation with sera from immunized mice (Fig. 5B). This finding suggests that the adhesion capacity of EHEC is decreased *in vitro*.

It is worth mentioning that sera obtained from mice immunized with the empty P22 capsid (Cp+adj) caused a significant reduction (*P < *0.01) in the adherence of EHEC to epithelial cells. This was an unexpected result; however, it is plausible to hypothesize that nonspecific E. coli proteins remained attached to the viral capsids or were trapped inside them during the purification process. This could elicit antibodies that recognize EHEC antigens other than EscC and that could inhibit adherence to epithelial cells. Another possibility is that nonspecific antibodies directed to the capsid also inhibited adherence.

**FIG 6:**
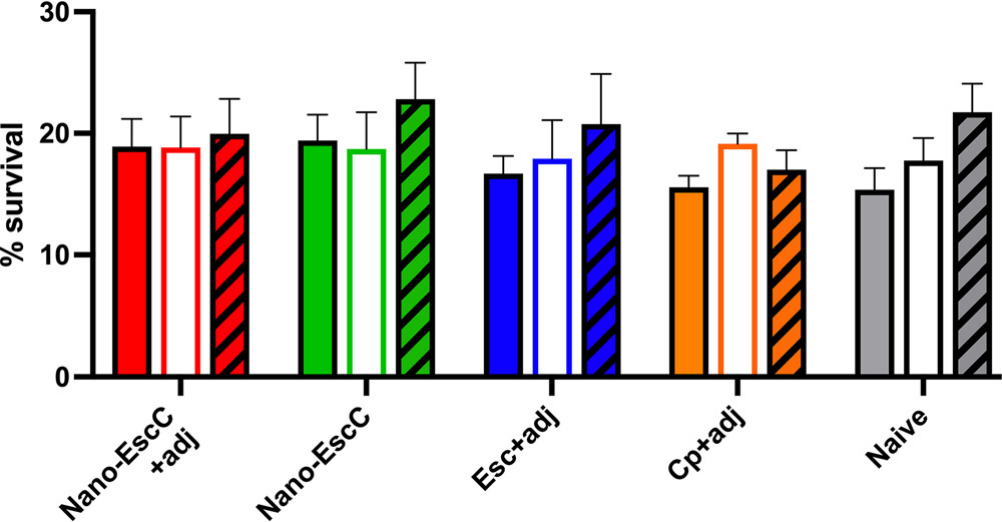
Bactericidal activity of serum from mice vaccinated with different formulations or unvaccinated mice (naive). In each group, colored bars represent the active sera, white bars the inactive, and the striped bars show the inactive sera supplemented with mouse sera as an exogenous complement source. The survival percentage was established as follows: bacteria recovered/input × 100. Results are expressed as means of two independent experiments measured in triplicate + SEM.

Finally, we evaluated whether the serum from the immunized mice had bactericidal activity. For this, we quantified the percentage of EHEC survival in the presence of active serum, inactive serum, or inactive serum supplemented with a complement source, to evaluate the activity by complement-mediated destruction. However, we found no significant differences among conditions or groups (Fig. 6).

Taken together, these complementary assays support the notion that Nano-EscC-generated antibodies might be functional but that antibodies generated against CPs alone might interfere with the functional characterization. Among the alternatives that would allow to obtain better results than the main approach is increasing the loading capacity of the nanocontainers by improving the EscC/Cp ratio, triggering a more specific immune response. Alternatively, functionalizing the viral capsid with the same encapsulated protein (e.g., EscC) would also increase the specific immune response against the antigenic protein. Another possibility is to include other adjuvants to increase the immune response, such as the immunostimulant 3-O-desacyl-4-monophosphoryl lipid A (MPLA) (44), its synthetic analog RC529 (45), or AFPL1, an immunostimulant that induces a high protective immune response based on a Th1 pattern, which provokes cellular and humoral responses, with a good level of safety (46). Overall, our data demonstrated that these nanocontainers can function as a delivery model of these antigens by using self-assembled viral capsids from the P22 phage.

### Conclusions.

Through the *in vivo* and *in vitro* data obtained, we conclude that nanovaccines utilizing P22 VLPs as carriers for EHEC antigens have the potential to elicit a specific immune response against EHEC. It is important to consider the confinement capacity of P22-based nanocontainers for the formulation of nanovaccines. It is essential that the number of antigenic proteins as cargo be as large as possible. An alternative to improve the antigenic capacity is to functionalize the outer surface of the capsid proteins with the antigenic proteins, and thus increase the amount of antigen present in the formulation. The production of nanocontainers with higher loads of antigenic proteins, the possible functionalization of their outer surface, as well as increasing the number of immunizations, could generate more effective nanovaccines against different pathogenic bacteria.

On the other hand, intranasal immunization offers advantages such as ease of self-administration and induction of mucosal immunity. Self-administration is easily done by intranasal drop or spray administration; it is not invasive and causes little discomfort to subjects, among other advantages.

Due to the ease of production and low cost of these VLP-based nanovaccines, they might be an alternative vaccine platform attractive to low-income or middle-income countries. Another no less important aspect to consider is that the proteins of the viral capsid elicit an immune response. Previous studies demonstrated the immunogenicity of P22 capsids and their possible role in increasing the immune response against their cargo proteins, without the need to add adjuvants (41). Our results suggest that the capsids generate an immune response that can interfere with the one produced by the nano-EscC against EHEC, since its production was conducted in the same bacterial species. The protein nature of the viral capsid, its bacterial origin, as well as its high percentage in relation to the cargo protein (EscC) resulted in significant nonspecific antibody production, so the production of nanocontainers with maximum load is essential for improving the production of nanovaccines. The manufacture of nanovaccines based on P22 could have greater potential if they are developed against microorganisms other than E. coli, which would avoid cross-reactions or contamination that would interfere with the production of neutralizing antibodies, or the purification protocol can eliminate all the contaminants. P22-nanovaccines are viable, simple, safe, effective, and low-cost alternatives for the development of vaccines against diverse types of infectious agents.

## MATERIALS AND METHODS

### Bacterial strains and growth conditions.

The EHEC O157:H7 86-24 strain was used in this study. Bacteria were routinely grown overnight aerobically at 37°C with agitation at 200 rpm in Luria-Bertani (LB) broth. For epithelial cell infections, bacteria were also incubated in the presence of Dulbecco’s minimum essential medium (DMEM) without supplementation for 2 h to induce the expression of type 3 secretion system components, as previously described (12).

### EHEC protein expression and P22-based nanovaccine production.

EHEC *escC* (encodes EscC T3SS structural protein), *lomW* (encodes outer membrane protein), and *eae* (encodes intimin) genes were amplified by PCR using the Q5 High-Fidelity DNA Polymerase (New England Biolabs), EHEC O157:H7 86-24 genomic DNA as the template, and the primers listed in Table 1.

**TABLE 1:** Primers used in this work

Primer name	Sequence 5′ to 3′	EHEC target gene
Fw escC-NcoI	gatataccatggccatgaaaaaaataagtt	*escC*
Rv escC-XhoI	tgctactcgagccttcgctagatgcagat	
Fw lomW-NcoI	gatataccatggccatgaagagtatagcaac	*lomW*
Rv lomW-XhoI	tgctactcgaggaatttcaggccaatg	
Fw eae-NcoI	gatataccatggccatgattactcatggttg	*eae*
Rv eae-XhoI	tgctactcgagttctacacaaaccgca	

The restriction sites for NcoI and XhoI were introduced flanking each amplified gene. The genes were cloned into the vector pBAD *ansB-sp* after double digestion (39), replacing the *ansB* gene with each one of the EHEC genes (*escC*, *lomW*, or *eae*), resulting in the vectors pBAD *escC-sp*, pBAD *lomW-sp*, and pBAD *eae-*sp. The selected plasmids were transformed into the chemically competent E. coli BL21(DE3) bacteria and selected in LB ampicillin-supplemented media accordingly (47). Constructs were verified by restriction analysis with NcoI and XhoI enzymes in agarose gel, and by DNA sequencing using the UTMB DNA core. Overexpression of fusion proteins (EscC-SP, LomW-SP, or Intimin-SP) was induced by 0.125% L-arabinose (Sigma-Aldrich). Subsequently, the pRSF/P22-CP vector, encoding the bacteriophage P22 coat protein, was transformed by electroporation into E. coli BL21(DE3) cells harboring each one of the pBAD constructions previously described (pBAD *escC-sp*, pBAD *lomW-sp*, or pBAD *eae-sp*). Cotransformants were then selected by ampicillin and kanamycin resistance. Overexpression of P22-CP protein was induced with 0.5 mM isopropyl β-D-1-thiogalactopyranoside (IPTG) (Sigma). All products were confirmed by SDS-PAGE (12%), as reported by Sánchez-Sánchez et al. (48).

In order to obtain P22-based VLPs, differential expression of both fusion proteins and CP protein was performed as described (49). Briefly, cells were grown in LB broth supplemented with 50 μg/mL kanamycin (Sigma-Aldrich), and 200 μg/mL ampicillin (Sigma-Aldrich) to maintain plasmids at 37°C and 180 rpm until reaching an absorbance of 0.9 at 600 nm. Then, fusion protein expression was induced by adding 0.125% L-arabinose at 30°C and 180 rpm for 16 h. Afterward, P22-CP expression was induced by adding 0.5 mM IPTG (Sigma) for 4 h under the same culture conditions. For P22 empty capsid production, the pRSF/P22-CP was transformed into E. coli BL21(DE3) cells, overexpressed after induction with 0.5 mM IPTG, and product confirmed by SDS-PAGE (12%). The cultures were centrifuged for 15 min at 8,000 × *g*, and bacterial pellets were kept at −80°C until use.

For protein identification, once size was confirmed by SDS-PAGE gels, samples were submitted to the Mass Spectrometry Facility (UTMB) for further identification. Briefly, samples were prepared using standard proteomics sample preparation conditions. Because MALDI-TOF was not sufficient for identification, LCMS was run and data were analyzed using Proteme Discovere 2.5 software, with the sequest node used for peptideID and the minora node for Label-Free Quan (LFQ) using the MSpeak areas for each of the peptide-spectral matches (PSMs).

### Purification of P22 capsids and P22-based nanovaccines.

P22 capsids and EscC-P22, LomW-P22, and Intimin-P22 nanovaccines were obtained by sonicating bacterial pellets in PBS buffer (pH 7.6) for 20 min (10 s on, 10 s off) in an Ultrasonic processor. After centrifugation, the supernatants were recovered and then underwent ultracentrifugation in PBS and 35% sucrose cushion for 1 h at 41,000 × *g* and 4°C in an Optima XPN-100 centrifuge (Beckman Coulter). The pellets were resuspended in 4 mL PBS (pH 7.6) under constant and slow agitation.

P22 capsids or nanovaccines were then purified using a HiPrepTM 16/60 Sephacryl S-500 HR column (Cytiva) and an AKTA prime plus fast protein liquid chromatography (FPLC) (GE Healthcare). Fractions containing the P22 capsids or P22-based nanovaccines were pooled, concentrated using Amicon Ultra centrifuge filters 100 MWCO (Millipore), verified by SDS-PAGE, and stored at 4°C for further characterization. Endotoxin levels were assessed using a Pierce LAL chromogenic endotoxin quantification kit (ThermoFisher Scientific) following instructions of the manufacturer. The fusion proteins–CP protein ratio was determined for each formulation by densitometric analysis of protein bands separated by SDS-PAGE gels using NIH Image J software (version 1.53t).

### TEM characterization.

P22 capsids, EscC-P22, LomW-P22, and Intimin-P22 nanovaccines for TEM observations were obtained by placing 10 μL of diluted dispersions (~0.10% wt/wt) onto a copper grid (400 mesh, Formvar/carbon, TedPella). Samples were stained with 2% uranyl acetate (ThermoFisher Scientific), air dried, and analyzed with a JEOL JEM-2010 TEM (Tokyo, Japan) operated at 200 kV (UTMB Microscopy Core). To observe the size, shape, and internal density of the nanocontainers, micrographs were taken at different magnifications.

### EscC expression and purification.

EscC expression was induced from a pET30a/*escC* expression vector and purified as previously described (24). Briefly, overnight cultures of BL21(DE3)-competent E. coli cells (New England BioLabs) containing a pET30a (+) harboring the *escC* gene encoding EscC were diluted 1:20 in LB broth. Cultures were grown until log phase and induced with 1 mM (final concentration) IPTG. Four hours after induction, cultures were centrifuged (4,000 × *g* for 20 min), and resulting pellets were washed once with water and then frozen at −80°C. The bacterial pellets were thawed and resuspended in 20 mL lysis buffer (PBS containing 10% glycerol, 25 mM sucrose, 1 mg/mL final concentration lysozyme, and a tablet of cOmplete EDTA-free protease inhibitor cocktail [Roche, Germany]). The lysate was placed on ice for 30 min, sonicated, and centrifuged (16,000 × *g* for 30 min at 4°C) to pellet-insoluble material. To maximize soluble protein extraction, insoluble pellets were solubilized overnight at room temperature (RT) with 2% final concentration sodium dodecyl sulfate (SDS) in lysis buffer. Lysates were centrifuged, and the resulting pellet was used for subsequent solubilization. Soluble protein extracts were applied to detergent-removal spin columns to remove SDS (ThermoFisher Scientific), sterilized using a filter (0.2 μm pore size), and then bound to Talon metal affinity resin (TaKaRa Bio). The resin was washed with PBS containing 10 mM imidazole, and protein was eluted from affinity columns by applying PBS with 10% glycerol, 25 mM sucrose, and 150 mM imidazole. Pure fractions were collected and pooled before being dialyzed (Slide-A-Lyzer dialysis cassettes, ThermoFisher Scientific) overnight at 4°C in PBS with 10% glycerol and 25 mM sucrose. Endotoxin levels were assessed using a Pierce LAL chromogenic endotoxin quantification kit (ThermoFisher Scientific) following instructions of the manufacturer. Purified protein was run on an SDS-PAGE gel along with bovine serum albumin (BSA) standards, protein concentration was determined by bicinchoninic acid (BCA) protein assay and densitometric analysis using NIH Image J software (version 1.53t), and samples stored at −80°C until use.

### Mice immunization.

All animals in this study were handled in strict accordance with the recommendations in the Guide for the Care and Use of Laboratory Animals of the National Institutes of Health. The experimental protocol was reviewed and approved by the Institutional Animal Care and Use Committee of the University of Texas Medical Branch (protocol no. 1007037C). Female 6- to 8-week-old BALB/c mice (12 per group; Charles River) were used for immunization, as previously described (24, 25). Animals were housed in microisolator cages under pathogen-free conditions with food and water available *ad libitum* and maintained on a 12-h dark–light cycle. To allow adequate acclimation, mice were housed within the animal facility for 1 week before experimentation.

Prior to immunization, blood samples were taken retro-orbitally from each mouse to establish baseline levels. BALB/c mice were immunized intranasally twice 2 weeks apart with EscC-P22 nanovaccine. Each formulation contained 5 μg protein (EscC) in 50 μg P22-CP protein nanocontainer, with or without 10 μg detoxified cholera toxin (CT) as adjuvant (Sigma). Mice inoculated with P22 empty capsids plus adjuvant were used as a control group, as well as animals inoculated with 5 μg recombinant EscC protein plus adjuvant. Once mice were immunized, both appearance and physical activity were daily evaluated to confirm the lack of adverse reaction to inoculation. For that, different characteristics were evaluated: (i) behavior attitude: active, curious, or lethargic; (ii) normal stool or signs of diarrhea; (iii) normal fur or ungroomed appearance; (iv) normal breath or signs of dyspnea, rales, or tachypnea; (v) body weight. To evaluate antibody titers, blood was drawn retro-orbitally 14 days after the last immunization. To separate sera, blood was incubated for 30 min at RT to allow clotting and centrifuged at 5,000 × *g* for 5 min at 4°C. Sera were recovered and stored at −80°C for further use. At the end of the experiment, total sera and lungs were retrieved for storage and further investigation.

### Detection of protein-specific antibodies.

The protein-specific total IgG titers were determined by indirect enzyme-linked immunosorbent assay (ELISA), as previously described (50). Briefly, a high-binding 96-well microplate (Costar) was coated with purified EscC protein (1 μg/well) in Dulbecco’s phosphate-buffered saline (DPBS) (Corning) and incubated at 4°C overnight. Wells were washed three times with washing buffer (0.05% Tween 20 in DPBS) and then blocked with blocking buffer (0.05% Tween 20, 2% bovine serum albumin in DPBS) at RT for 2 h. The blocked wells were washed twice before the addition of sample diluent (1% BSA, 0.05% Tween 20 in DPBS). The sera were added to each top dilution well, and 2-fold dilutions were performed following incubation at RT for 2 h. Plates were washed three times with washing buffer. Next, diluted 1:5,000 horseradish peroxidase (HRP)-conjugate goat anti-mouse IgG (Southern Biotech) antibody was added into each well and then incubated for 3 h. Plates were washed four times before the addition of tetramethylbenzidine (TMB) substrate solution (Invitrogen, USA). After 15 min, stop solution (2N H_2_SO_4_) was added, and the samples were immediately read at 450 and 570 nm using a microplate reader (BioTek). The titers were reported as means ± standard deviations of optical density obtained from two independent experiments and compared to the baseline sera, and a multiple unpaired *t* test was used to establish significance. Endpoint titers were also established as mean reciprocal titers defined as mean plus twice the standard deviation of the levels measured for naive sera. Data are shown as mean ± standard error. Significance was evaluated by one-way ANOVA.

### Opsonophagocytic killing assay.

The opsonophagocytic ability was evaluated in murine macrophage cell line RAW 264.7 (ATCC TIB-71). Cells were routinely grown in complete DMEM (Gibco) cell culture medium (supplemented with 10% inactivated FBS, 1% sodium pyruvate, 1% nonessential amino acids, and penicillin/streptomycin 100 U/mL and 100 μg/mL, respectively), following the manufacturer’s instructions, and incubated at 37°C and 5% CO_2_. For infection analysis, 5 × 10^5^ cells/well were seeded in 24-well cell-culture-grade plates. EHEC O157:H7 strain 86-24 inoculum was used at a multiplicity of infection (MOI) of 10 (5 × 10^6^ CFU) were incubated in the presence or absence of heat-inactivated sera (30 min at 56°C) from immune groups (EscC-P22 nanovaccine with and without adjuvant, P22 capsids plus adjuvant, and recombinant EscC protein plus adjuvant) or naive sera (final concentration of 10%) for 1 h at 37°C, with slight agitation. After incubation, bacteria were collected in 1 mL of fresh medium and used to infect macrophage cell culture plates. Two hours after infection, intracellular bacteria were collected. To eliminate extracellular bacteria, cells were washed three times with PBS, and then, to obtain intracellular bacteria, cells were treated with 0.1% Triton X-100 (in PBS), serially diluted, and plated on LB for bacterial CFU enumeration. The intracellular survival was established as the percentage of recovered bacteria based on the inoculum for each group. Data represent the results of two independent experiments performed in triplicate using pooled sera from 12 mice per group. One-way ANOVA was performed. Quantitative data are expressed as means ± standard errors.

### Adherence inhibition assay.

Human intestinal epithelial cell line Caco-2 (ATCC HTB-37) was used to evaluate the bacterial adherence inhibition of immune sera. Cells were maintained at 37°C with 5% CO_2_ in complete DMEM (Gibco). For adhesion assays, 12-well plates were seeded at a confluence of 1 × 10^6^ cells/well. Overnight cultures of EHEC O157:H7 86-24 strain were diluted in DMEM (1:20) without supplements and incubated at 37°C under static growth conditions for 2 h, to induce T3SS components expression and adherence. Bacterial inocula were adjusted to an MOI of 10 (1 × 10^7^ CFU) and incubated in the presence of EscC-P22, P22, or EscC protein-inactivated (30 min at 56°C) immune sera for 1 h at 37°C with slight agitation or naive sera (final concentration 10%), as described above. After incubation in the presence of sera, bacteria were collected in 1 mL of fresh media (input) and used to infect the monolayer epithelial cells. After incubation for 2 h, cells were washed three times with PBS prior to the addition of 0.1% Triton X-100 (in PBS). Once detached, the cells were serially diluted in PBS and plated for enumeration of adhered bacteria (output). The percentage of adhered bacteria was determined as the output/input × 100. Data represent the results of two independent experiments performed in triplicate using pooled sera from 12 mice per group. One-way ANOVA was used for statistical analysis. Quantitative data are expressed as means ± standard errors.

### Serum bactericidal assay.

Serum bactericidal assays were performed as described previously (24). Briefly, sera from immunized or naive mice (*n* = 12/group) were pooled, and half of them were heat-inactivated (30 min at 56°C) as described above. EHEC O157:H7 strain 86-24 was grown for 16 h at 37°C in LB broth, diluted 1:20 in DMEM without supplements, and incubated statically for 2 h at 37°C (input). Then, bacterial cultures were either exposed to active, inactive (heat-inactivated), or mixed sera (inactive sera mixed 1:1 with naive sera from each group as a source of complement) at a final concentration of 10%, incubated for 1 h at 37°C with slight agitation (100 rpm), and plated onto LB agar for CFU enumeration after serial dilution (output). Two-way ANOVA was used for statistical analysis. Results are expressed as means of percent bacterial survival (output/input) with standard errors.

### Statistical analysis.

All statistical analysis was done using GraphPad Prism software (version 9.0). *P* values of 0.05 were considered statistically significant.
