# Supplementary material for: P22-Based Nanovaccines against Enterohemorrhagic Escherichia coli
Source: Microbiol Spectr. 2023 Mar 21;11(2):e04734-22. doi: 10.1128/spectrum.04734-22 (PMC10100862; doi:10.1128/spectrum.04734-22)
Supplement: Supplemental file 1 — Fig. S1 to S5 and Table S1. Download spectrum.04734-22-s0001.pdf, PDF file, 0.8 MB [file spectrum.04734-22-s0001.pdf]

SUPPLEMENTARY FIGURES.

P22-based nanovaccines against enterohemorrhagic *Escherichia coli*.

Alejandro Huerta-Saquero[1,2#*], Itziar Chapartegui-González[2#], Sarah Bowser[2], Nittaya Khakhum[2], Jacob L. Stockton[2], Alfredo G. Torres[2*]

[1] Centro de Nanociencias y Nanotecnología, Universidad Nacional Autónoma de México. Ensenada, B.C. México. 22860

[2] Department of Microbiology and Immunology, University of Texas Medical Branch. Galveston, Texas. USA. 77555

Running title: nanovaccines against *EHEC*

[#]These authors contributed equally to this work

*Co-corresponding authors, altorres@utmb.edu; saquero@ens.cnyn.unam.mx
Phone 409-747-0189
Fax 409-747-6869

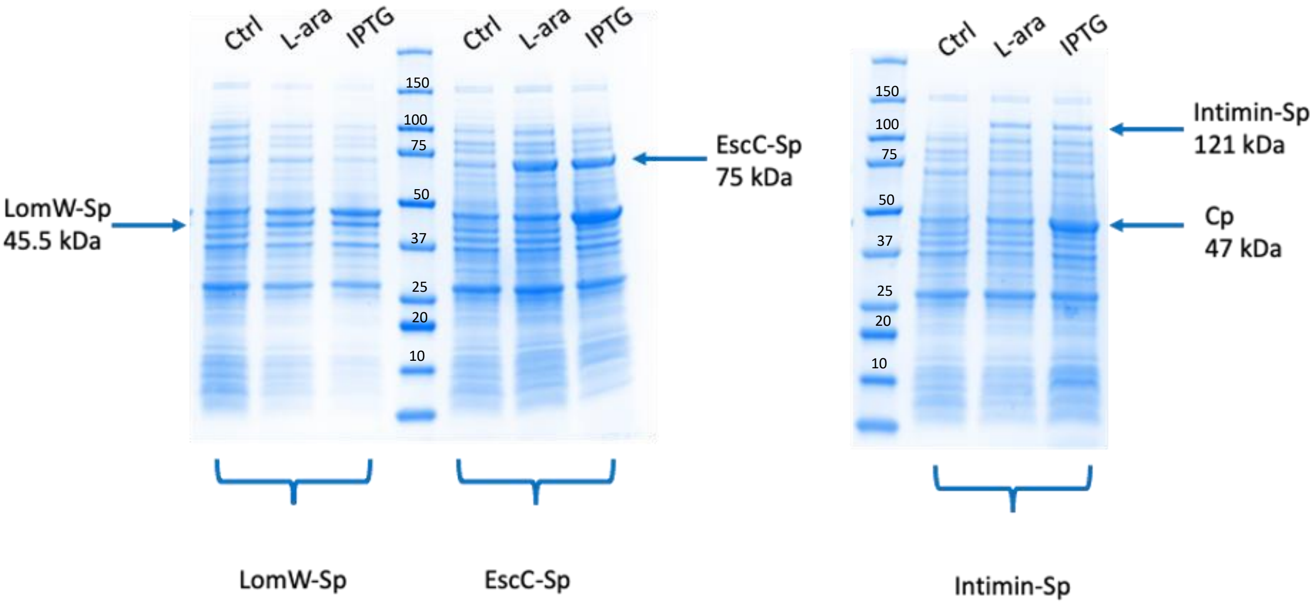

Supplemental Figure 1. SDS-PAGE of total proteins from BL21 *E. coli* cultures expressing LomW-Sp, EscC-Sp, Intimin-Sp, and CP proteins. Fusion protein expression was induced by adding 0.125% L-arabinose at 30°C and 180 rpm for 16 h. Next, P22-CP expression was induced by adding 0.5 mM isopropyl-β-D-1-thiogalactopyranoside (IPTG) for 4 h under the same culture conditions. Fusion and CP proteins are indicated by arrows.

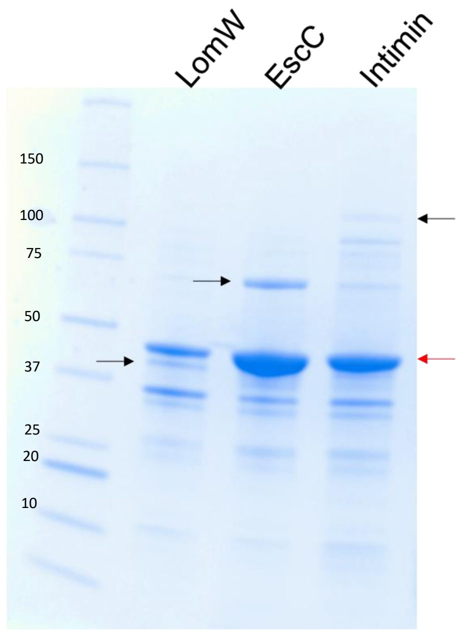

Supplemental Figure 2. P22-based nanovaccines purification. SDS-PAGE of nanovaccines after ultracentrifugation in 35% sucrose cushion and gel filtration chromatography in HiPrepTM 16/60 sephacryl S-500. Black arrows point to LomW-Sp, EscC-Sp and Intimin-Sp fusion proteins, respectively. CP protein is indicated by red arrow.

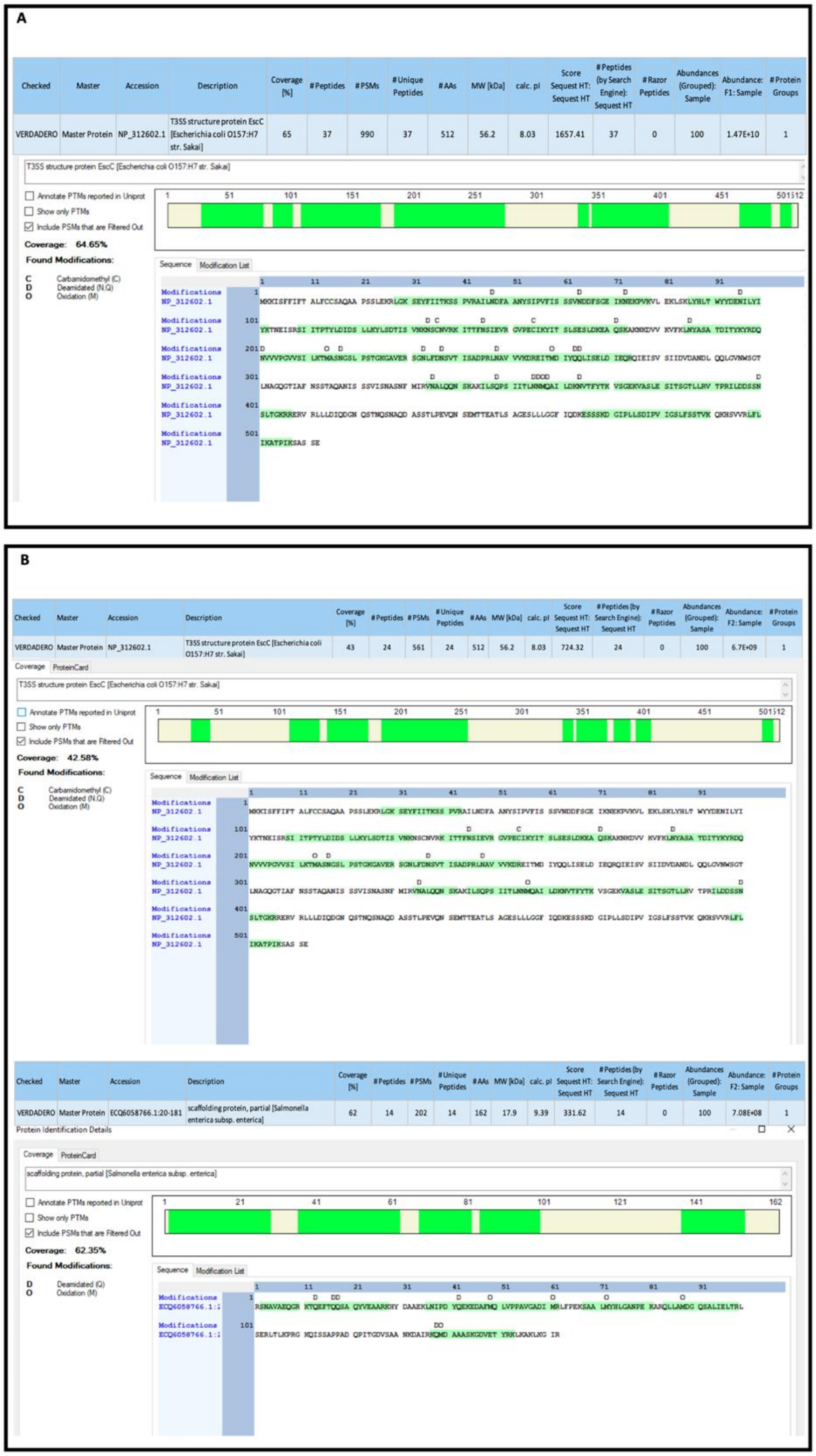

Supplemental Figure 3. Mass spectrometry protein sequencing results. **A**) EscC pure protein and **B**) EscC coupled with SP used for nanovaccines. It is shown the high abundant protein, as well as the maps of coverage of each of them. The normal contaminant proteins during processing were excluded.

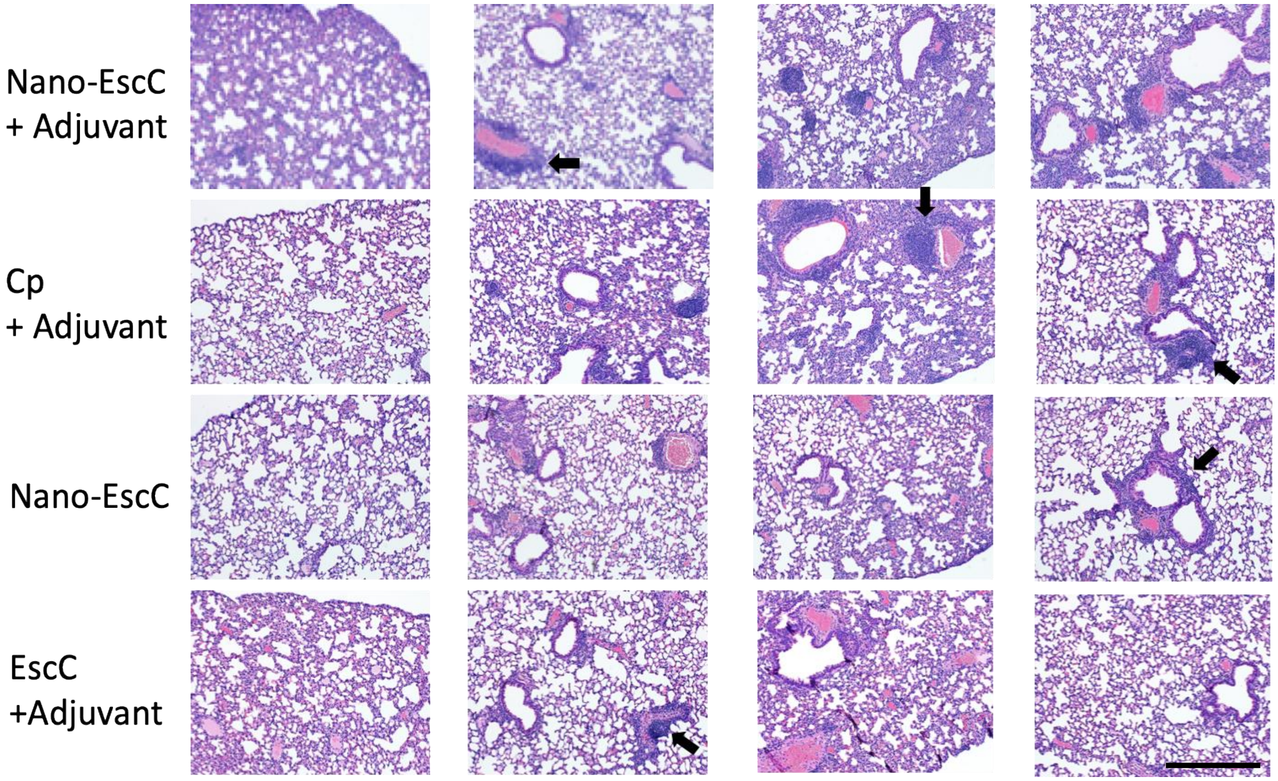

Supplemental Figure 4. Mice lung histology. Hematoxylin/eosin-stained sections of mouse lungs after immunizations at x400 magnification. No qualitative differences were found among immunized groups. The arrows indicate possible areas of inflammation. Scale bar, 100 µm.

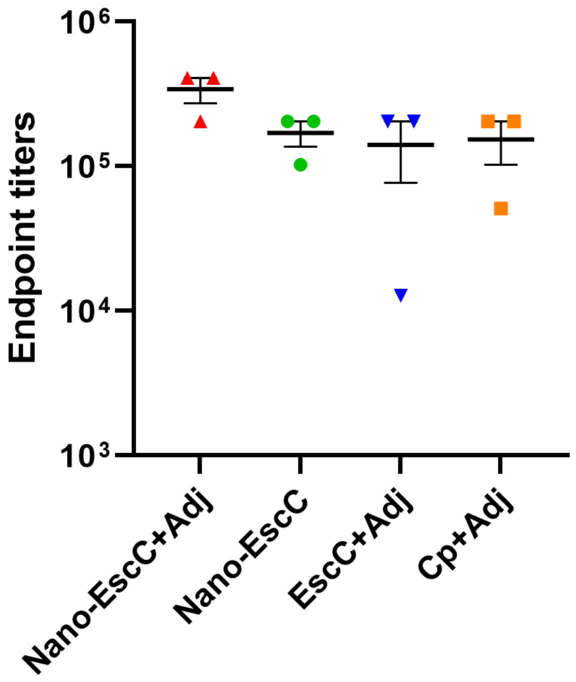

Supplemental Figure 5. IgG endpoint titers against EscC from EscC-P22 with and without adjuvant immunized mice were slightly higher compared to mice immunized with EscC+adjuvant and Cp+adjuvant. Although, no significant differences were obtained (One-way ANOVA).

Supplemental Table 1. Statistical significance from all the different dilutions tested compared with the baseline sera. Multiple unpaired *t*-test was used.

	**Nano-EscC *vs* Baseline**		**Cp+adj *vs* Baseline**		**Nano-EscC+adj *vs* Baseline**		**Esc+adj *vs* Baseline**	
**Dilution**	***p*-value**	**Significant**	***p*-value**	**Significant**	***p*-value**	**Significant**	***p*-value**	**Significant**
**1:800**	0.00077	Yes	0.00097	Yes	0.00066	Yes	0.00088	Yes
**1:1600**	0.00094	Yes	0.00121	Yes	0.00077	Yes	0.00098	Yes
**1:3200**	0.00113	Yes	0.00157	Yes	0.00084	Yes	0.00123	Yes
**1:6400**	0.00132	Yes	0.00145	Yes	0.00100	Yes	0.00197	Yes
**1:12800**	0.00131	Yes	0.00150	Yes	0.00136	Yes	0.00259	Yes
**1:25600**	0.00316	Yes	0.00246	Yes	0.00167	Yes	0.00288	Yes
**1:51200**	0.00451	Yes	0.00411	Yes	0.00243	Yes	0.01613	Yes
**1:102400**	0.01180	Yes	0.00763	Yes	0.00320	Yes	0.01010	Yes
**1:204800**	0.05132	No	0.05132	No	0.01005	Yes	0.02986	Yes
**1:409600**	0.29986	No	0.05654	No	0.16795	No	0.15485	No
